# Reliability of Visual Assessment of Non-Contrast CT, CT Angiography Source Images and CT Perfusion in Patients with Suspected Ischemic Stroke

**DOI:** 10.1371/journal.pone.0075615

**Published:** 2013-10-08

**Authors:** Tom van Seeters, Geert Jan Biessels, Joris M. Niesten, Irene C. van der Schaaf, Jan Willem Dankbaar, Alexander D. Horsch, Willem P. T. M. Mali, L. Jaap Kappelle, Yolanda van der Graaf, Birgitta K. Velthuis, on behalf of the Dust Investigators

**Affiliations:** 1 Department of Radiology, University Medical Center Utrecht, Utrecht, The Netherlands; 2 Department of Neurology, Utrecht Stroke Center, Rudolf Magnus Institute of Neuroscience, University Medical Center Utrecht, Utrecht, The Netherlands; 3 Department of Radiology, Rijnstate Hospital, Arnhem, The Netherlands; 4 Julius Center for Health Sciences and Primary Care, University Medical Center Utrecht, Utrecht, The Netherlands; University of Cambridge, United Kingdom

## Abstract

**Background and Purpose:**

Good reliability of methods to assess the extent of ischemia in acute stroke is important for implementation in clinical practice, especially between observers with varying experience. Our aim was to determine inter- and intra-observer reliability of the 1/3 middle cerebral artery (MCA) rule and the Alberta Stroke Program Early CT Score (ASPECTS) for different CT modalities in patients suspected of acute ischemic stroke.

**Methods:**

We prospectively included 105 patients with acute neurological deficit due to suspected acute ischemic stroke within 9 hours after symptom onset. All patients underwent non-contrast CT, CT perfusion and CT angiography on admission. All images were evaluated twice for presence of ischemia, ischemia with >1/3 MCA involvement, and ASPECTS. Four observers evaluated twenty scans twice for intra-observer agreement. We used kappa statistics and intraclass correlation coefficient to calculate agreement.

**Results:**

Inter-observer agreement for the 1/3 MCA rule and ASPECTS was fair to good for non-contrast CT, poor to good for CT angiography source images, but excellent for all CT perfusion maps (cerebral blood volume, mean transit time, and predicted penumbra and infarct maps). Intra-observer agreement for the 1/3 MCA rule and ASPECTS was poor to good for non-contrast CT, fair to moderate for CT angiography source images, and good to excellent for all CT perfusion maps.

**Conclusion:**

Between observers with a different level of experience, agreement on the radiological diagnosis of cerebral ischemia is much better for CT perfusion than for non-contrast CT and CT angiography source images, and therefore CT perfusion is a very reliable addition to standard stroke imaging.

## Introduction

Modern imaging techniques have important additional value in the diagnostic workup of patients with acute ischemic stroke. A combination of non-contrast CT (NCCT), CT angiography (CTA) and CT perfusion (CTP) parameters can provide information on (early) ischemic signs and the extent of the ischemic changes, which helps to establish the diagnosis and may guide treatment decisions [1], [2], [3], [4], [5].

Frequently used methods to estimate the extent of early ischemic changes are the 1/3 middle cerebral artery (MCA) rule and the Alberta Stroke Program Early CT Score (ASPECTS). The 1/3 MCA rule can be used to assess quickly whether the ischemic area is larger or smaller than 1/3 of the total supply territory of the MCA [6], whereas ASPECTS is a more extensive and time consuming score that allocates points for ischemic changes in specific areas of the MCA territory [7], [8]. Both methods were initially developed for use on NCCT, but are now increasingly used for the assessment of ischemia on CTA source images (CTA-SI) and CTP [9], [10], [11], [12], [13], [14], [15], [16].

The underlying pathophysiology of ischemic changes on NCCT, reflecting cytotoxic edema, is different from CTA and CTP, reflecting brain perfusion. However, all three imaging modalities can be available in the early diagnostic work-up of ischemic stroke patients. Therefore, one simple and robust method to evaluate all three modalities is desirable. Preferably, such a method should also be reliable when performed by observers with less experience (i.e. radiology residents), since residents will often need to evaluate NCCT, CTA and CTP in the acute setting outside office hours without direct supervision of an expert. Until now the reliability of the 1/3 MCA rule and ASPECTS for application on CTA-SI and CTP in the acute setting remains unclear.

Our aim was to perform a reliability study for the 1/3 MCA rule and ASPECTS on different CT imaging techniques in patients suspected of acute ischemic stroke, by assessing the inter- and intra-observer agreement of both methods on NCCT, CTA-SI and CTP, between observers with varying experience.

## Methods

### Ethics Statement

The medical ethics committee of the University Medical Center Utrecht approved the study. Because stroke patients may have a compromised ability to consent (e.g. due to a decreased level of consciousness or aphasia), both the treating neurologist and the investigator established whether patients had the capacity to consent. If this was the case, written informed consent was obtained from the patients themselves. In patients with a compromised ability to consent, written informed consent was obtained from their nearest relative(s). Patients who die before informed consent can be obtained are an exception. Because it is undesirable to burden the relatives with this request, the medical ethics committee waived the need for informed consent in these patients.

## Patients

All patients are participants of a large, prospective multicentre observational cohort study of patients with suspected ischemic stroke. On admission, all patients underwent neurological examination, NCCT, CTP and CTA. Inclusion criteria were: a) acute neurological deficit of less than 9 hours duration, suspected to be caused by ischemic stroke; b) National Institutes of Health Stroke Scale (NIHSS) of at least 2; and c) 18 years or older. Exclusion criteria were: a) bleeding or other diagnosis than ischemic stroke on NCCT; and b) known contrast material allergy or renal failure. Patients who awakened with stroke symptoms could only be included if the time from going to sleep until imaging was less than 9 hours.

For this study, we included all consecutive patients with a suspected acute ischemic stroke from 4 participating hospitals for whom imaging evaluation was performed between January 2011 and September 2011. Patients were excluded if image quality was poor or if either of the image slices required for ASPECTS were missing in the CTP slab.

### Imaging Protocol

All imaging studies were performed on multislice CT scanners. Depending on the hospital, they were performed on either a Philips 128-detector scanner, a Philips 64-detector scanner, a Philips 40-detector scanner, or a Toshiba 64-detector scanner. Patients underwent NCCT first, then CTP, and finally CTA.

NCCT was performed using 120 kV, 300–375 mAs, and slice thickness 5 mm. CTP coverage was at least 40 mm and covered the basal ganglia up to the lateral ventricles to ensure that both ASPECTS levels were included. Forty ml of non-ionic contrast material was injected intravenously with a flow of 6 ml/s followed by 40 ml of saline with a flow of 6 ml/s. Images were acquired every 2 seconds for 50 seconds after initiation of contrast injection. CTP scans were performed with 80–120 kV and 150–200 mAs adjusted to local scanner, and reconstructed as 5 mm contiguous axial slices. For the CTA, from aortic arch to cranium vertex, 50–70 ml of non-ionic contrast material was injected intravenously with a flow of 6 ml/s followed by 40 ml of saline with a flow of 6 ml/s. The scan delay after intravenous contrast injection was calculated for each patient individually from time to peak arterial enhancement on CTP.

### Post-processing of CTP

Post-processing was performed on standard, clinical available CTP software (Extended Brilliance Workstation, version 4.5, Philips Medical Systems) to calculate cerebral blood volume (CBV) and mean transit time (MTT) maps. To obtain predicted penumbra and infarct maps, we used previously reported MTT and CBV thresholds [17]. The total ischemic area was defined as a relative measure of MTT ≥145% compared to the contralateral (unaffected) hemisphere. Within this ischemic area, infarct was separated from penumbra by an absolute value of CBV <2.0 ml/100 g [17].

### Assessment of Imaging

All scans were assessed on a clinical workstation (Extended Brilliance Workstation, version 4.5, Philips Medical Systems) by two observers from a pool of six: one of two neuroradiologists (I.v.d.S. and B.V.) and one of four radiology residents (T.v.S., J.N., J.W.D., and A.H.). The neuroradiologists each had more than 10 years of experience in assessment of NCCT and CTA, and 6 years in assessment of CTP. The residents had between 1 and 4 years of experience in assessment of all imaging techniques. The observers were blinded for all clinical information, except side of symptoms. To avoid bias during evaluation, all scans were assessed in the same order: NCCT, CTA-SI, CBV maps, MTT maps, and eventually penumbra and infarct maps. As this study focuses on reliability of assessment, we use the term ischemia in the broader sense to include both probable irreversible ischemic damage (infarct) and potentially reversible ischemia (penumbra).

On NCCT, an area was considered ischemic if there was parenchymal hypoattenuation with or without swelling of the brain [8], [18]. Areas with isolated cortical swelling, but without hypoattenuation, were not considered early ischemic changes [18]. Standard window level/window width settings were used (40/80). Observers were encouraged to change these standard settings to maximise contrast between normal and ischemic brain tissue [19]. CTA-SI were evaluated using reconstructed 5 mm average images, and areas with diminished contrast enhancement were considered ischemic [9]. For CTA-SI, observers used narrow window width to show maximum contrast between normal and ischemic brain tissue (window length/window width 50/50), and were allowed to manually change these settings. On CTP, areas were considered ischemic if there was a reduction in CBV or increase in MTT, compared to the contralateral hemisphere [11], [13]. For penumbra and infarct maps, the aforementioned thresholds were used.

**Figure 1:**
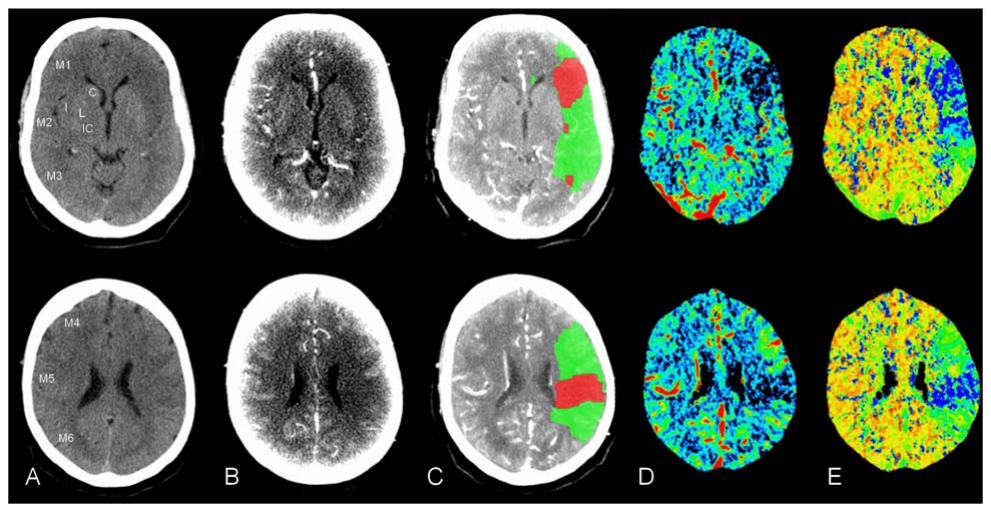
NCCT, CTA-SI and CTP in a patient with acute stroke. A 67-year-old male with aphasia and right-sided hemiparesis. Imaging approximately 1 hour after symptom onset. The upper row corresponds to the ganglionic ASPECTS level (C, caudate nucleus; L, lentiform nucleus; IC, internal capsule; I, insular region; M1–M3, cortical regions), the lower row to the supraganglionic ASPECTS level (M4–M6, cortical regions). Ischemic signs in the left MCA territory are seen on (A) NCCT; (B) CTA-SI; and on CTP in (C) penumbra and infarct maps; (D) CBV maps; and (E) MTT maps.

First, each scan was assessed for presence of ischemia both inside and outside the MCA territory (including posterior circulation). Second, the 1/3 MCA rule was applied and ASPECTS was determined (Figure 1). For the 1/3 MCA rule, the extent of ischemic changes was estimated visually to be more or less than 1/3 of the MCA territory [6]. For ASPECTS, two standardised levels of the MCA territory were evaluated: one at the level of the basal ganglia (ganglionic level), and one rostral to the ganglionic structures (supraganglionic level). On these levels, one point was allotted for ischemic signs in each of the following ten areas: caudate nucleus, lentiform nucleus, internal capsule, insular region, and 6 cortical regions (M1–M6). Then, the number of points was subtracted from 10 (no ischemic signs) to calculate the final ASPECTS [7], [8], [18]. For patients with no ischemic changes or only ischemic changes outside the MCA territory, the 1/3 MCA rule was scored as <1/3, and no points were scored for ASPECTS (i.e. ASPECTS = 10).

For intra-observer agreement, four observers evaluated twenty randomly chosen scans twice, blinded for their first assessment. To avoid recall bias, the minimum time period required between two assessments was two weeks.

### Statistical Analysis

Descriptive statistics were used for baseline characteristics. McNemar’s chi-square was used to determine differences in detection of ischemia across the different modalities. We used kappa statistics and intraclass correlation coefficient (ICC) to calculate inter- and intra-observer agreement. For the 1/3 MCA rule we used an unweighted kappa, as it is a dichotomous variable. Because ASPECTS is an ordinal 10-point scale, and since near agreement is better than far disagreement, we used a squared weighted kappa for ASPECTS. We considered kappa and ICC values <0.20 as poor, 0.21–0.40 as fair, 0.41–0.60 as moderate, 0.61–0.80 as good, and 0.81–1.00 as excellent. All statistical analyses were performed with R version 2.12.0 [20]. To calculate the (weighted) kappa statistics and intraclass correlation coefficient, we used the kappa2 function and icc function from the interrater reliability (irr) package [21].

## Results

Of the 116 patients who fulfilled the inclusion criteria, 2 were excluded because image quality was too poor, and 9 were excluded because one of the two ASPECTS slices was not included in the CTP slab. The remaining 105 patients were used for the analyses. In 100 patients (95.2%) the final discharge diagnosis was ischemia. Fourteen of these patients (14.0%) recovered completely within 24 hours. The remaining 86 patients (86.0%) had an ischemic stroke, of whom 14 patients (16.3%) had a lacunar infarct, and 16 (18.6%) had a posterior circulation stroke. The majority of patients (78.1%) was examined within 4.5 hours after symptom onset. Additional baseline characteristics can be found in Table 1.

**Table 1:** Baseline characteristics.

Number of patients		105
Age (years), mean ±SD		65.5±14.3
Male gender		57 (54.3)
NIHSS, median (IQR)		6 (3–12)
Time of symptom onset to admission	0–4.5 hours	82 (78.1)
	4.5–6 hours	15 (14.3)
	6–9 hours	8 (7.6)
Hypertension		64 (61.0)
Diabetes		15 (14.3)
Smoking status	Current smoker	28 (26.7)
	Ex-smoker	25 (23.8)
Hypercholesterolemia		38 (36.5)
Stroke in medical history		19 (18.1)
Final diagnosis	Ischemic stroke	86 (81.9)
	TIA	14 (13.3)
	Non ischemic	5 (4.8)

All values are given as number (%), unless otherwise indicated.

SD, standard deviation; IQR, interquartile range.

### Detection of Ischemia

Presence of ischemia in any brain region, also outside the MCA territory, was detected much more frequently with CTP than with either NCCT or CTA-SI (p<0.001 for all CTP maps compared to NCCT and CTA-SI). On NCCT, both observers indicated in 14 patients (13.3%) that ischemic changes were present, and on CTA-SI in 16 patients (15.2%, p = 0.773). On CTP maps, both observers indicated in 39 patients (37.1%) that ischemia was present on CBV maps; in 61 patients (58.1%) on MTT maps; in 58 patients (55.2%) on penumbra maps; and in 44 patients (41.9%) on infarct maps. Kappa and ICC values for agreement in detection of ischemia in any brain region are presented in Table 2.

**Table 2:** Detection of ischemia^a^ (n = 105).

	Inter-observer agreement		Intra-observer agreement	
CT modality	κ	ICC	κ	ICC
NCCT	0.541	0.543	0.604	0.623
CTA-SI	0.568	0.569	0.641	0.669
CTP CBV	0.805	0.810	0.900	0.900
CTP MTT	0.900	0.905	0.838	0.839
CTP penumbra maps	0.922	0.925	0.896	0.897
CTP infarct maps	0.904	0.904	0.850	0.851

a Dectection of ischemia inside and outside the MCA territory (including posterior circulation).

### Inter-observer Agreement 1/3 MCA Rule and ASPECTS

For NCCT, inter-observer agreement was moderate (κ = 0.428 and ICC = 0.431) for the 1/3 MCA rule and fair to good (κ = 0.219; ICC = 0.601) for ASPECTS (Table 3). For CTA-SI, it was poor for the 1/3 MCA rule (both κ and ICC = 0.168) and moderate to good for ASPECTS (κ = 0.583; ICC = 0.755). In contrast, inter-observer agreement for CTP was excellent for both the 1/3 MCA rule (range κ = 0.820 to 0.955; ICC = 0.823 to 0.956) and ASPECTS (range κ = 0.852 to 0.980; ICC = 0.930 to 0.980).

**Table 3:** Inter-observer agreement for 1/3 MCA rule and ASPECTS (n = 105).

	1/3 MCA rule		ASPECTS	
CT modality	κ	ICC	κ	ICC
NCCT	0.428	0.431	0.219	0.601
CTA-SI	0.168	0.168	0.583	0.755
CTP CBV	0.820	0.823	0.971	0.971
CTP MTT	0.865	0.868	0.929	0.930
CTP penumbra maps	0.955	0.956	0.980	0.980
CTP infarct maps	0.835	0.837	0.852	0.970

### Intra-observer Agreement 1/3 MCA Rule and ASPECTS

For NCCT, intra-observer agreement was poor (both κ and ICC = −0.079) for the 1/3 MCA rule and moderate to good (κ = 0.595; ICC = 0.622) for ASPECTS (Table 4). For CTA-SI, it was fair for the 1/3 MCA rule (κ = 0.267; ICC = 0.281), and moderate for ASPECTS (κ = 0.518; ICC = 0.567). For CTP however, intra-observer agreement was good to excellent for the 1/3 MCA rule (range κ = 0.707 to 0.920; ICC = 0.714 to 0.920), and was excellent for ASPECTS (κ = 0.915 to 0.967; ICC = 0.917 to 0.967).

**Table 4:** Intra-observer agreement for 1/3 MCA rule and ASPECTS (n = 80).

	1/3 MCA rule		ASPECTS	
CT modality	κ	ICC	κ	ICC
NCCT	−0.079	−0.079	0.595	0.622
CTA-SI	0.267	0.281	0.518	0.567
CTP CBV	0.707	0.714	0.933	0.934
CTP MTT	0.918	0.919	0.966	0.966
CTP penumbra maps	0.920	0.920	0.967	0.967
CTP infarct maps	0.861	0.864	0.915	0.917

## Discussion

In this study we investigated the reliability of two frequently used methods for imaging of acute ischemic stroke between observers with a different level of experience. Our results show that detection of ischemia with the 1/3 MCA rule and ASPECTS is easier and more reliable with CTP than with NCCT or CTA-SI. Both rating methods have excellent inter-observer agreement when used on CTP, but is much lower when used on NCCT and CTA-SI. In addition, intra-observer agreement is much better for CTP than for NCCT and CTA-SI. Evaluation of CTP therefore seems to ‘narrow the gap’ between neuroradiologists and residents, which is apparent for the evaluation of NCCT and CTA-SI.

Although reliability of the 1/3 MCA rule and ASPECTS has been investigated previously, most studies only investigated reliability on NCCT [6], [7], [8], [22], [23], [24], [25], [26], or only determined inter-observer agreement [6], [7], [8], [11], [12], [22], [23], [24], [26]. To the best of our knowledge, reliability of the 1/3 MCA rule on CTA-SI and CTP has never been investigated before, while reliability of ASPECTS for CTA-SI and CTP has been investigated previously [11], [12], [27]. Most previous studies analyzed patients who were treated with, or fulfilled clinical criteria for, thrombolysis, instead of all patients suspected of acute ischemic stroke in the emergency department [6], [7], [8], [11], [12], [23], [24]. In order to apply the 1/3 MCA rule or ASPECTS to CTP or CTA-SI for making treatment decisions in the acute setting, information about the reliability of both methods is necessary in all patients suspected of ischemic stroke, including patients whose final diagnosis is not ischemic stroke (e.g. patients with transient ischemic attack, migraine, or another stroke mimic). Although differences in study population between our study and previous studies make it difficult to compare previous findings with ours, our result for the 1/3 MCA rule on NCCT is quite comparable with previous literature (κ = 0.36–0.59) [6], [7], [8], [22], [23], [24], [25]. However, our κ-value for ASPECTS on NCCT is somewhat lower than previously reported (κ = 0.34–0.82) [7], [8], [22], [26]. One important reason for this difference is probably the experience of the observers. While most studies use experienced neuroradiologists and neurologists, we felt it was important to include residents with experience as they will need to make the decisions in the acute setting during off-office hours when treatment should be given as fast as possible. NCCT has the lowest contrast discrimination, is the most difficult modality to learn, and is the study in which experience is the most valuable.

Surprisingly, CTA-SI are more difficult to assess than we expected, especially for the 1/3 MCA rule. Possible reasons for this unexpected finding might be less experience with CTA-SI, and the gradually decreasing enhancement in the boundaries of the ischemic area on CTA-SI (compared to CTP), leading to difficulties in interpreting an area as >1/3 or <1/3 of the MCA territory. Furthermore, CTA-SI performed by modern multidetector CT scanners correlate better with CBF than CBV, since modern scanners are too fast to achieve a situation with arterial and tissue contrast steady-state during scan acquisition [28]. Hence, the hypoattenuated area on CTA-SI from modern scanners is somewhat larger than the true infarct core, which correlates best with CBV [14]. However, in our study acquisition of CTA-SI was after CTP, and therefore CTA-SI were obtained with a preload of CTP contrast. Consequently, arterial and tissue contrast steady state existed during acquisition of CTA-SI, with CTA-SI shifting more towards CBV weighted and a smaller hypoattenuated area.

Our study shows CTP as easy to read and reliable, which is very important in the acute setting. Although MRI diffusion weighted imaging (DWI) is thought to be reliable in assessing acute infarction, it is not always available and feasible in the acute setting and will certainly not facilitate increasing treatment of all stroke patients within and beyond the 4.5 hours. Hence, the role of CT in the management of acute stroke patients will remain important. Our data show that the reliability of ASPECTS for CTP parameter maps is similar to that of DWI [29].

In the present study, we assessed the inter- and intra-observer agreement of the 1/3 MCA rule and ASPECTS as a measure of precision. Other aspects of both methods, such as accuracy (which would require a reference standard) and prognostic value (which requires data on clinical outcome), will have to be evaluated in further studies.

Our study has some limitations. First, the MCA territory was not completely included in the CTP slab, which had a coverage of 40–65 mm. Nevertheless, we excluded patients if either one of the ASPECTS levels was not included in the CTP slab, and therefore only the most cranial part of the MCA territory was not visible on CTP. We think it is unlikely that ischemic changes we could have missed on CTP, would have affected the 1/3 MCA rule and ASPECTS much. Second, we performed a serial assessment instead of an independent evaluation of NCCT, CTA-SI and CTP. This sequential design of imaging assessment may have biased the imaging interpretation to some extent, as the observers have knowledge of the NCCT during evaluation of CTA-SI, and know both NCCT and CTA-SI during evaluation of CTP. However, in our view this approach only partially reflects clinical practice, as in stroke imaging NCCT is always available prior to additional CTA or CTP. Furthermore, since ischemia was detected much more frequently on CTP than on CTA-SI, a possible bias is probably relatively small. A more general limitation is that the κ-statistic can be affected by a low prevalence of the condition under study [30], which might (partially) explain the slightly negative intra-observer κ-value we found for the 1/3 MCA rule on NCCT, since in our study few patients have >1/3 MCA territory affected on NCCT. Another general limitation is that posterior circulation ischemia is not taken into account by both the 1/3 MCA rule and ASPECTS.

## Conclusion

Between observers with a different level of experience, assessment of ischemia with the 1/3 MCA rule and ASPECTS is easier and more reliable with CTP than with NCCT and CTA-SI. Therefore, CTP is a very reliable addition to NCCT and CTA in stroke imaging. Definite diagnostic and prognostic value of CTP has to be evaluated in subsequent studies.

## References

[1] Wintermark M, Meuli R, Browaeys P, Reichhart M, Bogousslavsky J, et al (2007) Comparison of CT perfusion and angiography and MRI in selecting stroke patients for acute treatment. Neurology 68: 694–697. 17325279 10.1212/01.wnl.0000255959.30107.08

[2] Schaefer PW, Barak ER, Kamalian S, Gharai LR, Schwamm L, et al (2008) Quantitative assessment of core/penumbra mismatch in acute stroke: CT and MR perfusion imaging are strongly correlated when sufficient brain volume is imaged. Stroke 39: 2986–2992. 18723425 10.1161/STROKEAHA.107.513358

[3] Muir KW, Halbert HM, Baird TA, McCormick M, Teasdale E (2006) Visual evaluation of perfusion computed tomography in acute stroke accurately estimates infarct volume and tissue viability. J Neurol Neurosurg Psychiatry 77: 334–339. 16239323 10.1136/jnnp.2005.074179 PMC2077700

[4] Tan JC, Dillon WP, Liu S, Adler F, Smith WS, et al (2007) Systematic comparison of perfusion-CT and CT-angiography in acute stroke patients. Ann Neurol 61: 533–543. 17431875 10.1002/ana.21130

[5] Lin K, Kazmi KS, Law M, Babb J, Peccerelli N, et al (2007) Measuring elevated microvascular permeability and predicting hemorrhagic transformation in acute ischemic stroke using first-pass dynamic perfusion CT imaging. AJNR Am J Neuroradiol 28: 1292–1298. 17698530 10.3174/ajnr.A0539 PMC7977671

[6] von Kummer R, Allen KL, Holle R, Bozzao L, Bastianello S, et al (1997) Acute stroke: usefulness of early CT findings before thrombolytic therapy. Radiology 205: 327–333. 9356611 10.1148/radiology.205.2.9356611

[7] Barber PA, Demchuk AM, Zhang J, Buchan AM (2000) Validity and reliability of a quantitative computed tomography score in predicting outcome of hyperacute stroke before thrombolytic therapy. Lancet 355: 1670–1674. 10905241 10.1016/s0140-6736(00)02237-6

[8] Pexman JH, Barber PA, Hill MD, Sevick RJ, Demchuk AM, et al (2001) Use of the Alberta Stroke Program Early CT Score (ASPECTS) for assessing CT scans in patients with acute stroke. AJNR Am J Neuroradiol 22: 1534–1542. 11559501 PMC7974585

[9] Coutts SB (2004) ASPECTS on CTA Source Images Versus Unenhanced CT: Added Value in Predicting Final Infarct Extent and Clinical Outcome. Stroke 35: 2472–2476. 15486327 10.1161/01.STR.0000145330.14928.2a

[10] Camargo EC, Furie KL, Singhal AB, Roccatagliata L, Cunnane ME, et al (2007) Acute brain infarct: detection and delineation with CT angiographic source images versus nonenhanced CT scans. Radiology 244: 541–548. 17581888 10.1148/radiol.2442061028

[11] Parsons MW, Pepper EM, Chan V, Siddique S, Rajaratnam S, et al (2005) Perfusion computed tomography: Prediction of final infarct extent and stroke outcome. Ann Neurol 58: 672–679. 16240339 10.1002/ana.20638

[12] Aviv RI, Mandelcorn J, Chakraborty S, Gladstone D, Malham S, et al (2007) Alberta Stroke Program Early CT Scoring of CT Perfusion in Early Stroke Visualization and Assessment. AJNR Am J Neuroradiol 28: 1975–1980. 17921237 10.3174/ajnr.A0689 PMC8134254

[13] Lin K, Rapalino O, Lee B, Do KG, Sussmann AR, et al (2009) Correlation of volumetric mismatch and mismatch of Alberta Stroke Program Early CT Scores on CT perfusion maps. Neuroradiology 51: 17–23. 18787815 10.1007/s00234-008-0454-y

[14] Lin K, Rapalino O, Law M, Babb JS, Siller KA, et al (2008) Accuracy of the Alberta Stroke Program Early CT Score during the First 3 Hours of Middle Cerebral Artery Stroke: Comparison of Noncontrast CT, CT Angiography Source Images, and CT Perfusion. AJNR Am J Neuroradiol 29: 931–936. 18272553 10.3174/ajnr.A0975 PMC8128574

[15] Kloska SP, Dittrich R, Fischer T, Nabavi DG, Fischbach R, et al (2007) Perfusion CT in acute stroke: prediction of vessel recanalization and clinical outcome in intravenous thrombolytic therapy. European Radiology 17: 2491–2498. 17549483 10.1007/s00330-007-0696-9

[16] Eckert B, Kusel T, Leppien A, Michels P, Muller-Jensen A, et al (2011) Clinical outcome and imaging follow-up in acute stroke patients with normal perfusion CT and normal CT angiography. Neuroradiology 53: 79–88. 20422406 10.1007/s00234-010-0702-9

[17] Wintermark M, Flanders AE, Velthuis B, Meuli R, van Leeuwen M, et al (2006) Perfusion-CT assessment of infarct core and penumbra: receiver operating characteristic curve analysis in 130 patients suspected of acute hemispheric stroke. Stroke 37: 979–985. 16514093 10.1161/01.STR.0000209238.61459.39

[18] Puetz V, Dzialowski I, Hill MD, Demchuk AM (2009) The Alberta Stroke Program Early CT Score in clinical practice: what have we learned? International journal of stroke 4: 354–364. 19765124 10.1111/j.1747-4949.2009.00337.x

[19] Lev MH, Farkas J, Gemmete JJ, Hossain ST, Hunter GJ, et al (1999) Acute stroke: improved nonenhanced CT detection–benefits of soft-copy interpretation by using variable window width and center level settings. Radiology 213: 150–155. 10540655 10.1148/radiology.213.1.r99oc10150

[20] R Development Core Team (2011) R: a Language and Environment for Statistical Computing. Vienna, Austria: R Foundation for Statistical Computing.

[21] Gamer M, Lemon J, Fellows I, Singh P (2010) irr: Various Coefficients of Interrater Reliability and Agreement. R Package version 083.

[22] Mak HK, Yau KK, Khong PL, Ching AS, Cheng PW, et al (2003) Hypodensity of >1/3 middle cerebral artery territory versus Alberta Stroke Programme Early CT Score (ASPECTS): comparison of two methods of quantitative evaluation of early CT changes in hyperacute ischemic stroke in the community setting. Stroke 34: 1194–1196. 12690213 10.1161/01.STR.0000069162.64966.71

[23] Grotta JC, Chiu D, Lu M, Patel S, Levine SR, et al (1999) Agreement and variability in the interpretation of early CT changes in stroke patients qualifying for intravenous rtPA therapy. Stroke 30: 1528–1533. 10436095 10.1161/01.str.30.8.1528

[24] Marks MP, Holmgren EB, Fox AJ, Patel S, von Kummer R, et al (1999) Evaluation of early computed tomographic findings in acute ischemic stroke. Stroke 30: 389–392. 9933276 10.1161/01.str.30.2.389

[25] Dippel DW, Du Ry van Beest Holle M, van Kooten F, Koudstaal PJ (2000) The validity and reliability of signs of early infarction on CT in acute ischaemic stroke. Neuroradiology 42: 629–633. 11071432 10.1007/s002340000369

[26] Coutts SB, Demchuk AM, Barber PA, Hu WY, Simon JE, et al (2004) Interobserver variation of ASPECTS in real time. Stroke 35: e103–105. 15073381 10.1161/01.STR.0000127082.19473.45

[27] Finlayson O, John V, Yeung R, Dowlatshahi D, Howard P, et al (2013) Interobserver agreement of ASPECT score distribution for noncontrast CT, CT angiography, and CT perfusion in acute stroke. Stroke 44: 234–236. 23103490 10.1161/STROKEAHA.112.665208

[28] Sharma M, Fox AJ, Symons S, Jairath A, Aviv RI (2011) CT angiographic source images: flow- or volume-weighted? AJNR Am J Neuroradiol 32: 359–364. 21051518 10.3174/ajnr.A2282 PMC7965723

[29] Butcher K, Parsons M, Allport L, Lee SB, Barber PA, et al (2008) Rapid Assessment of Perfusion-Diffusion Mismatch. Stroke 39: 75–81. 18063829 10.1161/STROKEAHA.107.490524

[30] Feinstein AR, Cicchetti DV (1990) High agreement but low kappa: I. The problems of two paradoxes. J Clin Epidemiol 43: 543–549. 2348207 10.1016/0895-4356(90)90158-l

